# The Impact of Evolutionary Driving Forces on Human Complex Diseases: A Population Genetics Approach

**DOI:** 10.1155/2016/2079704

**Published:** 2016-05-30

**Authors:** Amr T. M. Saeb, Dhekra Al-Naqeb

**Affiliations:** Strategic Center for Diabetes Research, College of Medicine, King Saud University, P.O. Box 18397, Riyadh 11415, Saudi Arabia

## Abstract

Investigating the molecular evolution of human genome has paved the way to understand genetic adaptation of humans to the environmental changes and corresponding complex diseases. In this review, we discussed the historical origin of genetic diversity among human populations, the evolutionary driving forces that can affect genetic diversity among populations, and the effects of human movement into new environments and gene flow on population genetic diversity. Furthermore, we presented the role of natural selection on genetic diversity and complex diseases. Then we reviewed the disadvantageous consequences of historical selection events in modern time and their relation to the development of complex diseases. In addition, we discussed the effect of consanguinity on the incidence of complex diseases in human populations. Finally, we presented the latest information about the role of ancient genes acquired from interbreeding with ancient hominids in the development of complex diseases.

## 1. Introduction

Geneticists have made significant progress in understanding the genetics behind many human diseases. These accomplishments include monogenic disease such as Huntington's disease. On the other hand, the discovery of genetic determinants for complex diseases such as diabetes, Crohn's disease, ischemic heart disease, stroke and some types of cancer (e.g., lung, colon, prostate, and breast), schizophrenia, and bipolar disorder is still poorly understood [1, 2]. However, release of the complete human genome sequence in 2001 has improved our understanding of the patterns of human genome diversity and its linkage to human complex diseases in the last decade [3, 4]. In order to study genetic diversity of the human genome at population level, the HapMap project was initiated to investigate the genetic differences on both inter- and intrapopulation levels. This was made possible by the introduction of advanced technologies such as Chip-based genotyping and next-generation sequencing techniques [5–7]. All these efforts have led to a vast amount of population genetic information. For instance, allele frequencies and levels of genetic association information for 3.5 million single nucleotide polymorphisms (SNPs), allele frequencies of approximately 15 million SNPs, 1 million short insertions and deletions, and 20000 structural variants are now available [5–8]. This huge amount of genetic variation data has been used in many Genome Wide Association Studies (GWAS) on various human diseases. According to National Human Genome Research Institute, the number of published GWAS studies till May 28, 2014, is 1921 [9] focusing on different human traits, such as height (522), and diseases, such as diabetes (251), breast cancer (191), lung cancer (35), coronary heart disease (150), and hypertension (39). GWAS have generated vast amount of information that increased our understanding of the genetic basis of many complex diseases by identifying genetic variants associated with the disease and its distribution in different populations. The availability of this information facilitates deeper understanding of complex diseases in both population genetics and evolutionary context.

## 2. Origin of Genetic Diversity in Human Populations

There are several factors that determine the amount of inter- and intragenetic diversity in human populations, which in turn is reflected in different phenotypes, including healthy and diseased phenotypes. These include mutation rates and recombination events that create and reorganize genetic diversity on the molecular level. Moreover, other factors are capable of changing the population size such as migration rates in or out of the population and birth and death rates. In addition, cultural behavior of human populations, such as selective or directed marriages or consanguinity, is also capable of effecting allelic frequencies within populations [10–14].

Generally, genetic, historical, and archeological evidences supported the Out-of-Africa hypothesis, which emphasized the elevated diversity of the original African population [15–18]. On the other hand, other evidences suggest much more multifaceted scenario in which early human populations have interbred with ancient hominids such as Neanderthals and Denisovans that lead to 1–6% contribution in modern Eurasian genomes and Melanesian genomes [19–21].

## 3. Evolutionary Driving Forces Effecting Genetic Diversity

It is well known that the main driving forces of evolution in any population are mutation, natural selection, genetic drift, and gene flow. The ability of these driving forces to perform their role is dependent on the amount of genetic diversity within and among populations. Genetic diversity among populations rises from mutations in genetic material, reshuffling of genes through sexual reproduction, and migration of individuals among populations (gene flow) [22]. The effect of the evolutionary driving forces on genetic diversity and evolution depends on the amount of genetic variations that already exist in a population. The amount of genetic variation within a given population remains constant in the absence of selection, mutation, migration, and genetic drift [23].

## 4. New Environment Effect of Genetic Diversity

The migration of human populations to new and different geographical habitats with different environmental challenges such as new climate, food varieties, and exotic pathogens acted as selective pressure on human populations that lead to adaptive changes in population genetic makeup to cope with these new challenges in order to achieve the golden goal of survival [24]. This selective pressure “natural selection” leads to the increase of frequency of favored genetic makeups and the elimination of deleterious genetic makeups that fail to adapt with the new environmental challenges [25]. This in turn may lead to the reduction of genetic diversity. Thus, natural selective events have shaped the present genetic diversity of the existing populations and consequently genetic variants involved in many diseases in both direct and indirect fashion [26–30].

## 5. Genetic Differentiation among Human Populations and the Role of Gene Flow

Genetic differentiation among human populations is significantly influenced by geographical isolation due to the accumulation of local allele frequency differences [31]. It was Wright in 1943 that first introduced the theory of Isolation By Distance (IBD) which describes the accumulation of local genetic differences under the assumption of local spatial dispersal [32]. According to IBD theory, pairwise measures of genetic differentiation are expected to increase with increasing geographical separation. This was proven in human populations on global, continental, and regional scales [33–35]. Physical barriers such as mountain chains, deserts, and large water bodies can limit gene flow among populations. Limited migration of individuals or groups among population can have an effect on genetic diversity leading to genetic differentiation among these populations and leads to the adaptive evolution in isolation. For example, the Sahara barrier causes the north to south (N–S) major orientation of genetic differentiation among the inhabitants of Africa [31]. Another significant geographic barrier, which has been suggested as an obstacle for gene flow, is the Himalaya mountain range resulting in the east to west (E–W) pattern of Asiatic genetic differentiation despite the fact that many problems with human populations sampling around the mountain were documented [31, 36–38]. It is well known that the rate of genetic differentiation differs according to orientations in Africa, Asia, and Europe, but not in the Americas [31] which can partially be justified by the presence of physical barriers that limited gene flow in certain directions in these continents. Thus, lack of significant physical barriers justifies that lack of directional genetic differentiation in the two Americas.

It was found that when comparing two nearby populations, Europe was found to be the continent with the smallest genetic differentiation, in relation to geographic distances measured using *F*-statistics (FST) (FST = 5 × 10^−4^) followed by Asia (FST = 9 × 10^−3^), Africa (FST = 1.7 × 10^−2^), and America (FST = 2.6 × 10^−2^). Generally, the genetic differentiation among two European populations separated by a thousand km is at least one order of magnitude lesser than in African, American, or Asiatic populations [31].

## 6. Natural Selection: The Most Significant Evolutionary Driving Force

Negative selection, also called purifying selection, is the most well-known form of natural selection [39]. Negative selection removes disadvantageous alleles or mutations from the population gene pool and reduces their frequencies in the population with a reduction rate corresponding to their biological effect. Thus, we should expect that lethal, nonsynonymous, or nonsense mutations will be eliminated from the population gene pool faster than synonymous mutations. On the other hand, less deleterious mutations that have milder effect on the correct expression of a gene can be found in a lower frequency in the population. The resulting change of genetic diversity in the population gene pool is low since negative selection effect on these mutations is mild. Another form of natural selection is positive selection, also called Darwinian selection, in which natural selection favors genetic mutations that are advantageous for the fitness or the survival of individuals. Positive selection will increase the frequencies of such variants in the population gene pool [25, 40]. The increase of the frequencies of variants will affect the genetic diversity in the population directly and indirectly by increasing the frequencies of genetically linked variants through genetic draft or genetic hitchhiking process [41, 42]. For example, several data indicate that the 503F variant of* OCTN1* gene has increased in frequency due to recent positive selection and that disease-causing variants in linkage disequilibrium with 503F have hitchhiked to relatively high frequency, thus forming the inflammatory bowel disease 5 (IBD5) risk haplotype. Moreover, association results and expression data support IRF1, which is nearby of 503F hitchhiking variants, as a strong candidate for Crohn's disease causation [43]. This may justify the observation that IBD5, which is a 250 kb haplotype on chromosome 5, is associated with an increased risk of Crohn's disease in European population [44–46]. On the other hand, other genetic variants that are not linked with the positively selected variants will be eliminated resulting in reduction of genetic diversity in a process called selective sweep. For instance, evidences for positive selection at the* GPX1* locus (3p21) and recent selective sweep in the vicinity of the locus were observed in Asian populations [47].* GPX1* locus is a selenoprotein gene characterized by the integration of selenium into the primary sequence as the amino acid selenocysteine. Selenoproteins have antioxidant properties, and thus interindividual differences in selenoprotein expression or activity could encompass an effect on risk for a range of complex diseases, cancers, neurodegenerative disorders, and diabetes complications [48–51]. Information about selective sweep of* GPX1* gene can illustrate the role of selenoprotein genetic variants in the etiology of various human complex diseases [52–55]. An additional form of natural selection is the balancing selection, in which several alleles may coexist at a given locus if they are advantageous either individually or together [56, 57]. Balancing selection is favored when heterozygote genotype has a higher relative fitness than homozygote genotype. Crohn's disease and ulcerative colitis are examples of balancing selection mediated evolution, which have been shown to be evolved in response to pathogen-driven balancing selection [58]. Based on “hygiene hypothesis,” the lack of exposure to parasites in modern settings resulted in immune imbalances, augmenting susceptibility to the development of autoimmune and allergic conditions. Population genetics analysis showed that five interleukin (IL) genes, including IL7R and IL18RAP, have been a target of balancing selection, a selection process that maintains genetic variability within a population. Fumagalli et al. showed that six risk alleles for inflammatory bowel disease (IBD) or celiac disease are significantly correlated with micropathogen richness validating the hygiene hypothesis for IBD and provide a large set of putative targets for susceptibility to helminthes infections [58].

## 7. Detecting the Effects of Natural Selection

All mentioned above forms of selection create characteristic molecular fingerprint also called selection signature. These selection signatures could be in the form of differences in rate of nucleotide diversity, allele frequency spectra, haplotype diversity, or genetic differentiation within or among population genomes [59]. As mentioned above, the most famous method of detecting natural selection signature is FST which is depending on the level of genetic differentiation among populations who experienced diverse forms of selection pressures because of many reasons, such as geographical isolation and environmental or nutritional conditions [60, 61]. Thus geographical isolation along with varying selection forces should increase the degree of differentiation among human populations resulting in an increase in FST value at the locus under selection [62].

## 8. Natural Selection Signature on Complex Diseases in Human Populations

Natural selection signatures have been detected on many complex diseases (Table 1). Among the complex diseases showing clear signatures of natural selection among human populations is blood pressure. Genetic differentiation analysis (FST) of blood pressure associated single nucleotide polymorphism (SNP) analysis showed accelerated differentiation among the four studied European subpopulations, namely, Utah Residents with Northern and Western European ancestry (CEU), British in England and Scotland (GBR), Toscani in Italia (TSI), and Finnish in Finland (FIN), with FST (EUR) *p* value = 0.0022 and 0.0054, respectively, for systolic blood pressure (SBP) and diastolic blood pressure (DBP).

At the individual SNP level, a nonsynonymous SNP (rs3184504) in* SH2B3* gene that is associated with blood pressure showed significant differentiation between European and non-European populations with FST *p* value = 0.0042 and branch length *p* value = 0.0088. It was found that the allele (T) was rare in African and Asian populations with *q* = 0.03 and 0.01, respectively, while it has a high minor allele frequency of *q* = 0.47 in the European population [63]. Moreover, genome wide association (GWA) SNPs associated with systemic lupus erythematosus (SLE) showed the most significant collective molecular selection signatures among all studied inflammatory and autoimmune disorders. The 29 SLE SNPs were significant for global genetic differentiation among human populations with FST *p* value of 0.008 and branch length analyses *p* value of 0.0072. Most of the observed genetic differentiation in SLE associated SNPs allele frequencies differences was driven by differences between African and European populations with FST AFR-EUR *p* value of 0.0028 or the Eurasia split in the branch length analysis *p* value of 0.001. For instance, a risk SNP (rs6705628) identified in Asian samples had a low allele frequency in Europeans *q* of 0.01 but high allele frequency in Africans *q* of 0.36 and Asians *q* of 0.19 [63, 64]. In addition, the population genetics analysis of type 2 diabetes (T2D) suggested marginally increased differentiation of T2D SNPs among global populations with FST (ALL) *p* value of 0.0354, which was likely attributed to the Eurasia split from Africa. At the individual T2D SNP level, the rs8042680 in* PRC1* gene showed the most significant selection signal. This SNP has a high derived protective allele frequency in European but is rare in African and absent in Asian populations [63, 64]. An additional complex disease that showed selection signature is coronary heart disease (CHD). The population genetics analysis of CHD associated SNPs showed a marginal increase of genetic differentiation between African and European populations with FST (African-European (AFR-EUR)) *p* value of 0.034. The individual CHD SNP showing the most significant selection signal was rs599839 in PSRC1 gene, which was also significantly associated with low-density lipoprotein (LDL) [63, 65, 66].

Furthermore, several genetic differentiation analyses of GWA studies of SNPs associated with different types of cancers, such as breast, prostate, and colorectal cancers were performed. The most significant collective evidence of global population differentiations was observed in the 34 SNPs associated with prostate cancer with a global FST *p* value of 0.017 or total branch length *p* value of 0.01. Majority of the observed differentiation was mapped to the African lineage in the maximum likelihood (ML) branch length analysis *p* value (AFR) of 0.0002. The most two significant SNPs (rs1465618 and rs103294) are located in* THADA* gene and near* LILRA3* gene, respectively. Moreover, multiple SNPs (rs7590268, rs6732426, rs13429458, rs17030845, rs12478601, rs7578597, and rs10495903) in the* THADA* gene have been reported to be associated with a variety of complex traits or diseases such as cleft palate [67, 68], hair morphology [69], polycystic ovary syndrome [70, 71], platelet counts [72], type 2 diabetes [73], IBD, and Crohn's disease [74, 75]. This gene has also been reported as a gene under selection [30, 63, 76, 77]. In addition, a sign of high differentiation of colorectal cancer SNPs was detected among the three Asian populations, namely, Han Chinese in Beijing (CHB), Southern Han Chinese (CHS), and Japanese in Tokyo (JPT) with FST (ASN) *p* value of 0.0006. In addition, the significant colorectal cancer SNP rs4925386 in* LAMA5* gene has higher derived allele frequency in Africans, but relatively low frequencies in Asians and Europeans.

## 9. Natural Selection and Cancer

Even though [78] Peto et al. in 1975 suggested a paradox that advocated that large animals might have developed some mechanisms to resist cancer in a counterselection process [79], very few studies have investigated the effect of selection on the evolution of cancer-related genes. An example for cancer-related genes under negative selection is breast cancer 1, early onset gene (*BRCA1*) [39, 80]. Not only is this gene strongly associated with female breast cancer but its mutations have been reported as risk factor for several other types of cancers including male breast cancer, fallopian tube cancer, and pancreatic cancer [81–86]. On the other hand, signature of positive selection was identified on the TRPV6 gene, which is aggressiveness of prostate cancer among European-Americans. Additionally, TRPV6 gene has experienced positive selection in non-African populations, resulting in several nonsynonymous codon differences among individuals of different genetic backgrounds [87, 88]. Moreover* UGT2B4* gene, associated with increased risk of breast cancer in Nigerians and African Americans, shows molecular signatures of recent positive selection or balancing selection [89]. Furthermore, signature of positive selection was identified on the* PPP2R5E* gene, which is involved in the negative regulation of cell growth and division.* PPP2R5E* gene encodes a regulatory subunit of the tumor suppressing protein phosphatase 2A and resides in a naturally selected genomic region in the Caucasian population of the HapMap [90]. This observed positive selection favors the Caucasian population making them less susceptible for soft tissue sarcoma. Scrutinizing molecular signatures of selection of this gene can lead to the identification of disease susceptibility variants. This information shows that cancer disease and its related genes were under the forces of evolution and natural selection throughout the evolutionary history and these evolutionary forces worked differently in different human populations.

## 10. Detrimental Consequences of Historical Selection Events in Modern Time

It was suggested that prehistoric selection events that may favored some genetic variants in ancient lifestyles, such as hunter-gatherers lifestyle, are not advantageous any longer. On the contrary, these positively selected genetic variants have become disadvantageous in modern societies with modern lifestyles. Many complex diseases, such as diabetes, obesity, hypertension, inflammatory or autoimmune diseases, allergies, and cancers, may have been by-products of these disadvantageous prehistoric selection events that are not fit with modern and more sedentary lifestyles. An excellent example is the thrifty gene hypothesis and the evolution and increased incidence of diabetes in modern populations. The thrifty gene hypothesis was first suggested by Neel, who suggested that diabetes predisposition genotypes in modern times were advantageous genotypes historically [91]. These positively selected genotypes that favored the storage of large quantities of body fat and slower metabolic rates were advantageous in the nomadic hunter-gatherers lifestyle and expected famine incidences. However, the change in the lifestyle to more sedentary type and the increase of available food resources lead to high rates of obesity and increased the risk of developing type II diabetes in individuals carrying these genotypes at present. Several studies supporting thrifty genotype hypothesis showed that the rapid change to modern lifestyle has led to high risk of diabetes and high levels of obesity in studied populations such as Native Americans of the United States and Tongans of the Pacific populations [92, 93]. Nominal evidence for positive selection at 14 loci of the diabetes susceptibility in samples of African, European, and East Asian ancestry was found only when using locus-by-locus analysis [94]. However, the debate about the validity of thrifty gene hypothesis is still ongoing.

Additional examples of detrimental health costs of historical natural selection leading to nowadays complex diseases are inflammatory and autoimmune diseases, such as type 1 diabetes, inflammatory bowel disease, Crohn's disease, celiac disease, and rheumatoid arthritis. This can be justified by the hygiene hypothesis especially in North European populations [95]. The “hygiene hypothesis” was first proposed by Strachan [96]. The major concept of hygiene hypothesis is that coevolution with some pathogenic agents is protecting humans from a large spectrum of immune-related disorders. Historically, a strong and intensified immune response was the best way to survive in pathogen-rich environments; thus, it was under strong positive selection, despite the fact that the same pathogens are still present but advancement in hygienic care and the use of antibiotics and vaccination, in the modern societies, lead to the reduction of pathogen-driven selection pressures. This reduction of selection pressures led to the conversion of the intensified immune response from being advantageous for human survival to be a health burden through inflammatory and autoimmune diseases [95, 97]. There is an increase of prevalence of autoimmune diseases in both developed and developing countries compared to third world countries. For example, type 1 diabetes has become a serious public health problem in some European countries, especially Finland [98]. In addition, incidences of inflammatory bowel diseases, Crohn's disease or ulcerative colitis, and primary biliary cirrhosis are also rising. Similarly, Africans living in the United States and Asians living in the United Kingdom in these days exhibited a higher risk of developing allergic inflammatory diseases and asthma compared with the general population in these countries [99–101]. Genetic and ethnic backgrounds of these populations were found to have higher impact on the prevalence of asthma compared to environmental effects [102, 103]. Evolutionary justification of the above-mentioned examples is that, in these populations, alleles conferring high risk for inflammatory and autoimmune diseases were under strong selective pressure in the past and in different environmental conditions [104] and that inflammatory and autoimmune disorders observed nowadays are the by-products of past selection against infectious diseases [97].

## 11. Consanguinity and Complex Diseases

As we mentioned above, cultural behavior of human populations, such as directed marriages or consanguinity, is capable of effecting allelic frequencies and genetic diversity within populations. Complex diseases can be affected by consanguinity when they are controlled by multiple rare genes and transmitted in an autosomal recessive manner [105]. Unfortunately, little is known about the effects of consanguinity on the complex diseases despite its great importance to global health. It is worth mentioning that consanguineous marriage is a common tradition in many populations in North Africa, Middle East, West Asia, and South India [105, 106]. Highly consanguineous populations, especially those with relatively small effective population sizes, provide an uncomplicated route for identifying recessively inherited genes for complex diseases such as identifying multiple loci for Alzheimer disease in an Arab population [107]. Moreover, some studies showed increased incidence of complex diseases among consanguineous marriage offspring. For example, minimal but significant increase of schizophrenia incidence among progeny of cousin marriages among Bedouin Arabs was observed [108]. In addition, higher rate of ischemic stroke was observed among religiously isolated inbreeding population in Netherlands compared to the general population [109]. In addition, global high rate of consanguinity may have a special impact on a polygenic disease like diabetes mellitus, especially type 2 diabetes. Anokute, in a study of 210 cases of diabetes in the central region of Saudi Arabia, found that familial aggregation compared to nonaggregation yielded an odds ratio of 6 : 2, respectively, which suggests a casual association with diabetes that needs to be further explored in future studies [110]. These findings do not extend to other populations in the same region such as Palestinians and Bahrainis where there is no increase in prevalence of type 2 diabetes in consanguine marriages [111, 112]. A study by Bener et al., 2005, which was done in Qatar showed that diabetes was significantly common among the consanguineous marriages of the first-degree relatives compared with the control group (33.1% versus 24.6%) (OR = 1.59; 95% CI = 1.11–2.29; *p* = 0.008) [113]. In another study done in Qatar by Bener et al., 2007, to determine the extent and nature of consanguinity in the Qatari population and its effects on common adult diseases, the rate of consanguinity in the present generation was 51% with a coefficient of inbreeding of 0.023724 [114]. The consanguinity rate and coefficient of inbreeding in the current generation were significantly higher than the maternal rate (51% versus 40.3% and 0.023724 versus 0.016410), respectively. All types of consanguineous marriages were higher in this generation, particularly first cousins (26.7 versus 21.4% paternal and 23.1% maternal) and double first cousins (4.3 versus 2.9% paternal and 0.8% maternal). The current generation of consanguineous parents had a slightly higher risk for most diseases such as cancer, mental disorders, heart diseases, gastrointestinal disorders, hypertension, hearing deficiency, and diabetes mellitus. All the reported diseases were more frequent in consanguineous marriages. Gosadi investigated the potential effect of consanguinity on type 2 diabetes susceptibility in Saudi population [115]. He suggested that consanguinity might increase the risk of type 2 diabetes by earlier development of the disease and by strengthening possible genetic effect on fasting blood glucose (FBG). Contradictory results have been obtained from association studies on breast cancer in consanguineous populations for BRCA1 and BRCA2 genes [116, 117]. Though, valuable information about the genetic background of complex diseases can be obtained from consanguineous populations if cultural, religious, and political bias concerning consanguineous marriage are circumvented.

## 12. Ancient Genes and Complex Diseases

Neanderthals, ancient hominids, and modern humans have coexisted for thousands of years and interbred outside of Africa especially in Europe and Asia [17]. This leads to the presence of several Neanderthals ancient genes in current European and Asian genomes (approximately 1–4%), while no Neanderthals ancient genes were observed among current African populations [19, 118]. Moreover, it was found that Neanderthal component in non-African modern human was more related to the Mezmaiskaya Neanderthal (Caucasus) than to the Altai Neanderthal (Siberia) or the Vindija Neanderthals [118]. In addition, several studies showed a higher Neanderthal admixture in East Asians when compared to Europeans [12, 119–121]. It was found that genes affecting keratin were found to have been introgressed from Neanderthals into East Asian and European humans, suggesting Neanderthals donated both morphological adaptation genes modern humans to cope with the new environments outside of Africa [120, 121].

Moreover, recent studies showed that the increased rates of type 2 diabetes in Europeans and Asians compared to Africans are due to interbreeding with ancient Neanderthals. It was found that many genes associated with complex diseases such as systemic lupus erythematosus, primary biliary cirrhosis, Crohn's disease, and diabetes mellitus type 2 have been introgressed from Neanderthals into non-African modern humans [121]. Though some beneficial genes such as immune-related genes are donated from Neanderthal to non-African modern humans. For example, HLA-C ^*∗*^0702, found in Neanderthals, is common in modern Europeans and Asians but is rarely seen in Africans [122].

## 13. Conclusion

Population genetics and molecular evolution studies have paved the way to gain better understanding of genetic adaptation of human in order to cope with environmental and lifestyle changes. Understanding the effect of evolutionary driving forces on human complex traits, such as natural selection, facilitated our ability to understand the relationship between genetic diversity, adaptive phenotypes, and complex disease. Huge amount of population genetics data for different human populations is available and waiting to be investigated deeply integrating both population genetics and molecular evolution contexts. Molecular signatures of genetic variations such as single nucleotide polymorphism, copy number variation, and genomic structural variations should be investigated and linked with human adaptation, the changing environment, and complex diseases. In addition large scale investigations about changes in lifestyles and the development of complex diseases are needed, especially in the Arabian Gulf area where drastic lifestyle changes accrued after the petroleum discovery. Integrating information about population genetics, molecular evolution, environmental changes, epidemiology, and social and cultural studies is an immediate need. These multidisciplinary efforts can elucidate the relationship between molecular evolution concept and complex diseases and improve our understanding of the evolutionary mechanisms in disease susceptibility, resistance, or progression, in turn facilitating disease prevention, diagnosis, and treatment.

## Figures and Tables

**Table 1:** Description of natural selection signature on complex diseases in human populations.

Disease	Studied population	FST *p* value	References
Blood pressure	European populations (CEU, GBR, TSI, FIN)	(EUR) 0.0022 (SBP) 0.0054 (DBP)	[63]

Blood pressure	European, non-European (Africans and Asians)	rs3184504 = 0.0042	[63]

Systemic lupus erythematosus	African, Asian, European	Global = 0.008 AFR-EUR = 0.0028 Eurasia split = 0.001	[63, 64]

Type 2 diabetes	African, Asian, European	Global = 0.0354	[63, 123]

Coronary heart disease	African and European	AFR-EUR = 0.034	[63, 65]

Prostate cancer	African, Asian, European	Global = 0.017 AFR = 0.0002	[67, 68]

Colorectal cancer	Asian populations	ASN = 0.0006	[63]

CEU: Utah Residents with Northern and Western European ancestry, GBR: British in England and Scotland, TSI: Toscani in Italia, FIN: Finnish in Finland, EUR: European all SNPs FST *p* value, SBP: systolic blood pressure, DBP: diastolic blood pressure, and AFR: African.
